# Dynamic Allostery in the Methionine Repressor Revealed by Force Distribution Analysis

**DOI:** 10.1371/journal.pcbi.1000574

**Published:** 2009-11-20

**Authors:** Wolfram Stacklies, Fei Xia, Frauke Gräter

**Affiliations:** 1 CAS-MPG Partner Institute for Computational Biology, Key laboratory of Computational Biology, Chinese Academy of Sciences, Shanghai, China; 2 Max-Planck-Institute for Metals Research, Stuttgart, Germany; 3 Bioquant BQ0031, Heidelberg University, Heidelberg, Germany; National Cancer Institute, United States of America and Tel Aviv University, Israel

## Abstract

Many fundamental cellular processes such as gene expression are tightly regulated by protein allostery. Allosteric signal propagation from the regulatory to the active site requires long-range communication, the molecular mechanism of which remains a matter of debate. A classical example for long-range allostery is the activation of the methionine repressor MetJ, a transcription factor. Binding of its co-repressor SAM increases its affinity for DNA several-fold, but has no visible conformational effect on its DNA binding interface. Our molecular dynamics simulations indicate correlated domain motions within MetJ, and quenching of these dynamics upon SAM binding entropically favors DNA binding. From monitoring conformational fluctuations alone, it is not obvious how the presence of SAM is communicated through the largely rigid core of MetJ and how SAM thereby is able to regulate MetJ dynamics. We here directly monitored the propagation of internal forces through the MetJ structure, instead of relying on conformational changes as conventionally done. Our force distribution analysis successfully revealed the molecular network for strain propagation, which connects collective domain motions through the protein core. Parts of the network are directly affected by SAM binding, giving rise to the observed quenching of fluctuations. Our results are in good agreement with experimental data. The force distribution analysis suggests itself as a valuable tool to gain insight into the molecular function of a whole class of allosteric proteins.

## Author Summary

Proteins carry out most of the cellular processes, from metabolic reactions to the regulation and expression of genes. Tight and effective regulation of the executing protein machinery is commonly achieved by allostery. The only general requirement for allosteric communication is the transmission of a signal, e.g., the binding of a cofactor, from the ligand binding site to the allosteric (active) protein site; in other words an internal propagation of strain. Based on molecular dynamics simulations, we recently presented a method that allows visualization of force distribution in proteins. We here applied this method to MetJ, a transcription factor whose activity is regulated by a co-repressor. Interestingly, co-repressor binding does not cause visible structural changes, yet increases DNA binding affinity manyfold. We were able to reveal a network linking fluctuations of distal parts of MetJ, including the DNA binding interface. Mechanical strain caused by SAM binding propagates to certain key residues, thereby altering fluctuations and finally resulting in increased DNA binding affinity. By directly monitoring ligand induced strain, instead of conformational changes, which might be absent or slow, our force distribution analysis suggests itself suitable to detect the mechanically crucial motifs in allosterically regulated protein machineries.

## Introduction

Protein allostery plays a key role in the regulation of cellular functions such as transcription or enzymatic action [1]. It crucially governs the formation of protein or protein-DNA complexes as well as the functional activity of individual proteins. Allosteric signals used by nature are diverse, ranging from ligand binding to reversible covalent modifications such as phosphorylation, or changes in the environment like pH or temperature. Intriguing examples are allosteric proteins in which effector molecules bind distal to the active site [2],[3].

A fundamental question is how the allosteric perturbation is transmitted through the protein to the active site for functional regulation. Can we understand and predict the mechanism and the network of interactions that propagate an allosteric signal? Answering this question is a prerequisite for functional mutagenesis and rational design of allostery. Sequence-based statistical analysis has proven highly successful to detect signal propagation pathways within and between allosteric proteins on the basis of evolutionary constraints [4],[5]. On the theoretical side, various thermodynamic concepts for inter-domain communication in allosteric proteins have been established [6],[7]. As yet, the molecular basis for long-range allosteric coupling between the regulatory and active site of a protein remains a matter of debate. This is why a range of experimental and computational techniques to monitor conformational changes involved in allostery have been developed and applied [7],[8], among others NMR [9], molecular dynamics (MD) simulations [10],[11], normal mode analysis and elastic network models [12],[13].

The basic premise of the above approaches is a conformational transition between two distinct states or a shift in a pre-existing conformational ensemble upon allosteric perturbation. In a commonly accepted picture, allosteric signals cause a perturbation at the regulatory site of the protein, analogous to an externally applied force. The perturbation then dissipates as internal strain or energetic coupling through the protein to the active site [14]. Signal propagation in turn causes conformational rearrangements, inducing an enhancement or decrease in the protein's activity. However, examples of long-range allosteric communication in the absence of any obvious conformational changes [9],[15] question this picture, showing that allostery does not necessarily rely on a change in mean atomic coordinates. Instead, allosteric strain can dissipate through rigid scaffolds without detectable conformational rearrangements.

A more fundamental understanding of allostery would thus require a way to directly follow strain propagation through proteins. This could reveal the allosteric network in a protein even in the absence of - or prior to the occurrence of - conformational changes. We recently presented a method termed force distribution analysis (FDA), based on MD simulations, that allows to detect propagation of internal strain caused by an external signal through proteins. The high sensitivity of the method makes it possible to even detect propagation through stiff materials, where a signal will propagate causing only minimal conformational changes that are below the threshold of experimentally accessible resolution. We have previously demonstrated the feasibility of FDA to detect force propagation in two mechanically perturbed proteins, namely the highly robust titin immunoglobulin domain I27 [16], and silk crystalline units [17]. While classical approaches focus on conformational changes or ensemble redistributions as a consequence of the signal-induced strain, such as normal mode analysis or essential dynamics [18], FDA sets out from the strain distribution itself. This renders FDA a perfectly fitted tool to elucidate the mechanism underlying allosteric signaling in proteins in general, be it with or without the involvement of structural rearrangements.

**Figure 1:**
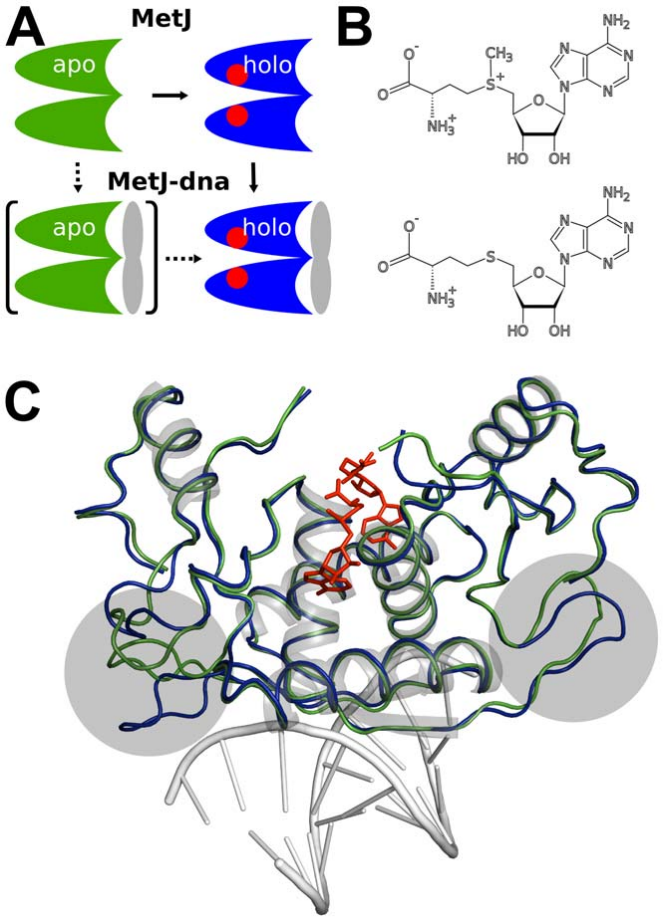
MetJ activation and SAM binding mode. (A) Schematic representation of MetJ activation. Binding of SAM increases the DNA affinity of MetJ manifold. (B) Chemical structure of SAM (top) and SAH (bottom). The molecules mainly differ in the positive charge on the sulfur atom. (C) Fit of crystal structures of MetJ in apo (1CMC, green) and holo (1CMB, blue) form. The only major difference between the structures is the conformation of the loops underlayed in gray. These loops are in direct contact with other MetJ molecules in the crystal lattice, and thus their conformation is unlikely to represent the true *in-vivo* configuration. The bound co-repressor SAM is shown in red.

We here chose to test the feasibility of FDA to detect allosteric networks in proteins using the classical textbook example of the methionine repressor protein MetJ [19]. MetJ is a challenging candidate, as it features long-range allosteric communication, yet without any noticeable changes in protein structure upon effector binding. MetJ is a transcription factor in the met regulon of *Escherichia coli*, the gene regulatory control system for methionine biosynthesis [20]. MetJ regulates the transcriptional levels of its own gene and those of several other proteins. Repressor activity results from binding to its operator, a specific 8 bp DNA sequence (the “metbox”), located in the promoter regions of genes regulated by MetJ. Changes in sequence of the metboxes are supposed to explain different regulatory activity [20],[21]. MetJ forms a homodimer in its native state [22]. In case of multiple adjacent metboxes it may form complexes of several homodimers arranged in a wheel-like structure around the DNA [23]. DNA binding of MetJ is regulated by its co-repressor, S-adenosylmethionine (SAM), an end product of methionine biosynthesis, Fig. 1A. Sensitivity for DNA is increased several-fold [24],[25] upon co-repressor binding. Of special interest is that SAM binds distant from the DNA binding site, with a minimal SAM-DNA distance of 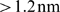 in crystal structures [26]. Holo and apo structures do not show significant structural changes [15]. For this reason it remains controversial how SAM influences DNA binding.

S-adenosylhomocysteine (SAH), a SAM analogue, binds MetJ with a binding affinity similar to SAM, but has no effect on its affinity for the operator (S. Philipps, Leeds Univ, 2009, personal communication). The main difference between SAM and SAH (Fig. 1B) is a positive charge on the sulfur atom of SAM, and it has been suggested that the increased sensitivity upon co-repressor binding is of purely electrostatic nature [27]. On the other hand, introduction of positive charges by a series of point mutations could not substitute the need for co-repressor [28]. Based on the force distribution pattern observed within the MetJ homodimer, we here propose a new model for MetJ activation upon cofactor binding. We measure directed propagation of internal strain from the SAM binding site to distinct residues in the DNA binding interface, through a specific network of a few key residues. The consequence is a wide-spread quenching of slow fluctuations and relocation and stiffening of specific side chains at the MetJ-DNA interface, leading to increased protein - DNA interaction. A distinct interaction pattern of individual residues with the co-repressor allows MetJ to fine-tune its response to co-repressor binding, explaining the inability of SAH to act as a co-repressor. Our results yield a molecular basis for MetJ allosteric function and are consistent with previous experimental studies.

## Results

### Molecular dynamics simulations

We carried out extensive MD simulations to elucidate the force distribution and conformational properties of MetJ. We used crystal structures of MetJ (PDB code 1CMC [15]) and MetJ in complex with DNA (PDB code 1CMA [26]) as starting point for our simulations. Throughout the manuscript, we will use the terms *MetJ* for the system without DNA and *MetJ-dna* for the MetJ-DNA complex. In both cases, simulations of the holo and apo forms were performed for comparison. Apo forms were created by deleting the bound SAM molecules from the crystal structures. An apo structure of MetJ is available, but as force distribution analysis is very sensitive to structural changes we decided to use the same crystal structure as basis for our simulations. Structures for Q44K, a mutant not relying on cooperativity to be functional [29], exist as well. Yet, as the altered charge distribution alters the DNA recognition pattern, though not the allosteric effect itself, we decided not to further investigate Q44K. For each of the five systems, 10 independent 30 ns MD simulations were performed, totaling 300 ns of simulation time, respectively. In agreement with crystallographic data [15], our simulations do not show major deviations between holo and apo forms. The overall backbone root mean square deviation (RMSD) of average structures is 0.66 Å for MetJ-dna and 0.64 Å for MetJ. This compares well with the crystal structures where we find a backbone RMSD for holo and apo structures of 1.63 Å which lowers to 0.59 Å after excluding poorly resolved loop regions having different conformation (residues 12–20 and 77–84), Fig. 1C. Crystal waters in the protein-DNA interface of 1CMA were found to quickly move into the bulk solvent and are thus unlikely to bridge specific interactions.

### Force distribution

To elucidate distribution of the allosteric signal induced by co-repressor binding, we directly calculated forces 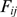 between each pair of atoms 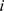 and 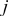 from our MD trajectories. We here analyze scalar pair-wise forces, which in contrast to the vectorial representation are unaffected by rotation of the system during the simulations. Observing pairwise forces has the advantage that forces do not average to zero over time, thus being the measure of choice for internal strain in systems equilibrated under a perturbation. Forces are calculated individually for bonded and non-bonded (electrostatic and van der Waals) interactions below the cutoff distance using the interaction potential defined by the Amber03 [30] force field. Long-range interactions as well as solvation effects such as screening of electrostatic forces and hydrophobic forces are not directly included in 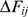, which is calculated only for the solutes and within the non-bonded cut-off. We however indirectly accounted for these effects by calculating forces from a system simulated in explicit solvent and with full electrostatics. Details are given in Methods. Propagation of the mechanical perturbation caused by SAM binding is measured as the difference in pairwise force, 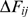, between the apo and holo forms of MetJ/MetJ-dna. For convergence, forces for each system were averaged over all ten equilibrium trajectories, each 30 ns in length. To reduce noise further, mainly resulting from slow side chain fluctuations that cannot equilibrate during simulation time, data were normalized as described in Methods. Dimensionless normalized changes in force are denoted 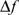.

The MetJ homodimer has a high degree of symmetry, and we thus expect the force distribution pattern to be highly symmetric as well. We checked this by calculating correlation coefficients between residue wise forces 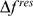, see Methods. Indeed, we find the force propagation pattern for the monomers to be very similar in all systems. For MetJ, residue wise forces correlate with 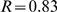, Fig. S2A. The MetJ-dna structure shows a less symmetric pattern, with 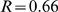, Fig. S2B. The lower symmetry of MetJ-dna might be a result of the lower resolution of the 1CMA crystal structure (2.8 Å for 1CMA vs. 1.8 Å for 1CMC) or of the only partially resolved DNA.

**Figure 2:**
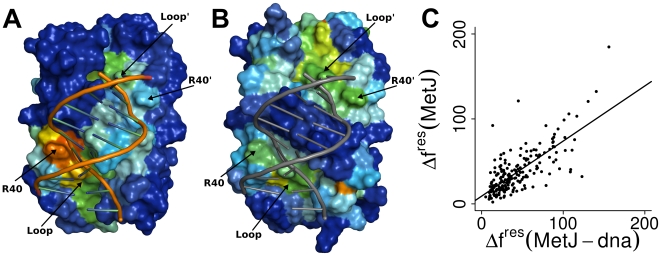
Force distribution at the protein - DNA interface. Force in (A) MetJ-dna and (B) MetJ is distributed to specifically targeted key residues on the protein-DNA interface. Only Arg40 and the loop formed by residues 50–53 show significant response to SAM binding. Large parts of helix A and the 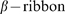 are not part of the allosteric regulatory mechanism. Colors for the protein surfaces range from blue for 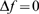 to red for high 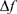; the DNA is displayed as sticks. For better overview, DNA was plotted into MetJ as well. (C) Correlations of changes in residue wise forces 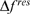 for MetJ and MetJ-dna. Both systems show a highly similar force distribution pattern, with a correlation of R = 0.74. The line displays the fit to a linear model.

Force distribution at the DNA binding site (Fig. 2A) reveals that remote MetJ binding induces a high degree of strain at distinct regions of the MetJ-DNA interface. In particular, Arg40 and a loop formed by residues 50–53 are subjected to high strain. The presence of the co-repressor thus is sensed by the DNA binding site, apparently via a long-range propagation of force from the bound SAM molecule through the protein scaffold to the MetJ-DNA interface. Importantly, the force distribution pattern was equally observed in the absence of DNA, Fig. 2B. In fact, forces in MetJ and MetJ-dna distribute in a very similar way, yielding a correlation of 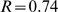, Fig. 2C. First, this is strong evidence that the observed change in forces is a result of SAM binding, independent from the presence of DNA. Second, as the initial crystal structures differ in resolution and conformation, the significant correlation highlights that the distribution pattern is robust with regard to the starting structure.

**Figure 3:**
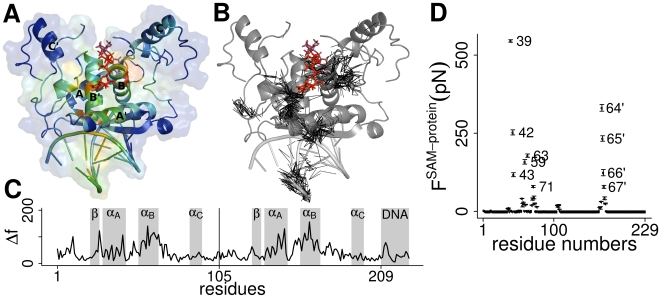
Force distribution in MetJ. (A) Changes in atomic forces, 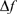, mapped onto a cartoon representation of the protein structure. Colors range from blue for elements outside the allosteric network with 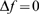 to red for force transducing elements with high 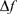. Helix identifiers are printed onto the structure. (B) Network-like representation of pronounced changes in inter-atomic forces observed upon SAM binding. Edges connect non-bonded atom pairs with 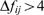. Forces between helix A and B are mainly propagated via side chain interactions. Propagation of the allosteric signal is highly anisotropic and directed, targeting individual residues at the protein-DNA interface while leaving large parts of the protein unaffected. (C) Changes in normalized pair-wise forces 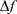 plotted along the MetJ sequence. The secondary structure is marked as gray bars. The vertical line indicates the start of the second monomer. (D) SAM interacts with a specific set of MetJ residues. Plotted are the forces exerted by SAM on MetJ. Numbers of strongly affected residues are plotted, residues in dimer 2 are marked with a stroke. Error bars show the standard error over the whole simulation time. Arg42 and Glu39 are among the most affected residues. Residues 64′–67′ are located far away from the binding site, close to the N-terminal end of helix B.

On the basis of FDA, we next investigated which protein structural elements are key to the strain distribution, allowing communication between SAM and the protein-DNA interface over a distance of more than 1 nm. Within the protein scaffold, we observe force propagation through helix B (B′) and forces are transmitted via side chain interactions onto helix A (A′), which in turn forms various side chain contacts with the DNA, Fig. 3A+B. Force propagation is highly non-isotropic and directed. This is to say, when compared to helix A and B, we see relatively little changes in pair-wise forces for the 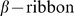 and the loops formed by residues 12–20, both in direct contact with the DNA, as well as for helix C (C′), Fig. 3C. In agreement with the low allosteric strain in the 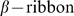, this motif, even though binding to the major groove of the metbox, has been found to play a role in DNA sequence specificity, but not in the allosteric regulation of DNA binding affinity [31]. Only a few side chains of helix A show significant changes in pair wise force, the strongest of which is observed for Glu39, Arg40, Arg42 and Arg43. Out of these residues only Arg40 is in direct contact with the DNA. This observation is remarkable as an almost complete loss of binding affinity was reported for mutation of Arg40 and its spacial neighbor Thr37, but not for mutation of others in direct contact with DNA [31]. Thr37, however, has been suggested to be involved in enhancing cooperativity, thereby only indirectly regulating DNA affinity. In agreement, we do not find Thr37 to be under SAM-induced strain.

We find two inter-related mechanisms of force propagation responsible for the specific targeting of the above mentioned structural elements. First, SAM strongly exerts a direct strain onto a set of MetJ residues, as reflected by extra-ordinarily high forces between the co-repressor and these residues, 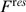, Fig. 3D. Most importantly, the adenosyl group of SAM strongly interacts with Glu39 and Arg42 in helix A, influencing their dynamics (see below and Fig. 3D).

**Figure 4:**
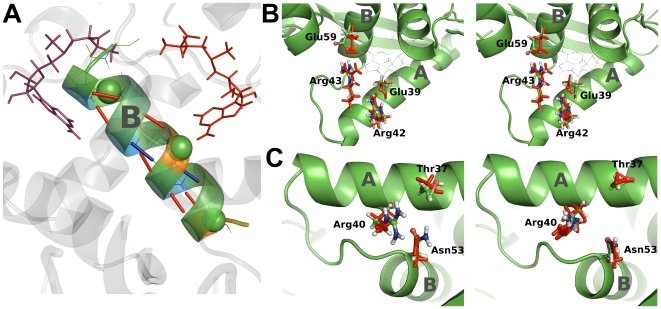
Subtle conformational changes induced by SAM binding. (A) Force distribution for backbone hydrogen bonds of helix B indicates helix bending. Hydrogen bonds are plotted as sticks, with red for increasing and blue for decreasing O-H Coulomb interaction. Spheres show the 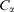 atoms of Ala64, Cys58 and Asn53. The angle between these atoms increases 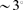 upon SAM binding. (B) Force transmission via a buried salt bridge and quenching of side chain fluctuations for MetJ-dna (left) and MetJ (right). Sticks display average coordinates over 300 ns in the apo (red) and holo (colors by atom type) configuration. Bending of helix B, supported by direct interaction with SAM, puts strain on the salt bridge formed by Glu59 and Arg43, visible as a small conformational rearrangement and high changes in pairwise forces. Fluctuations of Glu39 and Arg42 are quenched due to strong interaction with SAM, see also Fig. 4. (C) Relocation and stiffening of the Arg40 side chain for MetJ-dna (left) and MetJ (right). We measured tighter packing of the Thr37, Arg40 and Asn53 side chains and increased Arg40-DNA salt bridge formation.

Second, SAM features repulsive forces with helix B, inducing a high strain in the helix backbone hydrogen bonds. This apparently involves slight helix bending, Fig. 4A. Indeed, measuring the angle defined by the 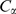 atoms of residues Ala64, Cys58 and Asn53 shows a bending upon SAM binding of 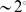 for MetJ and 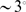 for MetJ-dna. We note that it is the significant difference in hydrogen bond forces, not in the mere atomic coordinates, between apo and holo form, that serves as robust indication for SAM-induced signal propagation. Helix bending in turn imposes strain on the salt bridge between Glu59 in helix B and Arg43 in helix A by minor conformational rearrangements, Fig. 4A+B. We measure high change in force 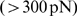 between these residues, suggesting this electrostatic interaction, buried in the protein core, to propagate force between helix B and helix A.

Both mechanisms, direct forces imposed from SAM onto key residues in helix A, and propagation of forces from SAM via bending of helix B, result in inconspicuous rearrangements at the DNA binding interface; most notably in the loop linking helices A and B (residues 50–53) and Arg40, as described above, Fig. 2A+B. Repositioning of Arg40 upon SAM binding is accompanied by an adjustment of the side chain packing with its direct neighbors, Thr37 and Asn53, Fig. 4C. Again, pairwise forces here served as a measure for signal propagation, rather than the only minor, yet reproducible coordinate changes (as for example a 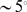 change of the angle in Asn53 between 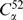, 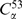 and 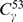 found for both, MetJ-dna as well as MetJ).

The described rearrangement of Arg40 caused by propagation of strain entails a strengthening of its saltbridge with DNA. From FDA, we measured an increase in attraction between Arg40 and DNA of 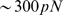. Overall, the potential energy between MetJ and DNA decreases by 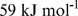 from 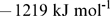 in the holo to 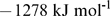 in the apo form, as a result of allosteric signaling by the co-repressor.

### The internal dynamics of MetJ

**Figure 5:**
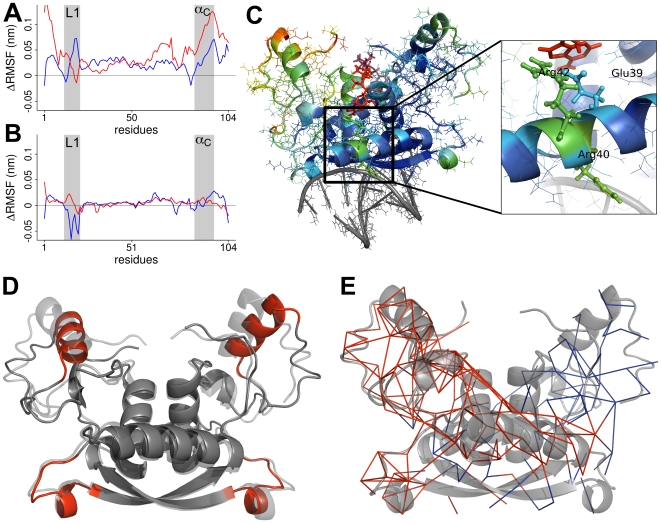
The dynamics of MetJ. (A, B) MetJ-dna (A) and MetJ (B) show quenching of fluctuations upon SAM binding. Plotted are differences in backbone root mean square fluctuations 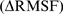 between apo and holo structures for both monomers (red and blue curves). Positive values indicate stiffening upon SAM binding. Loop 1 and helix C are underlaid in gray. Differences in 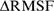 can be explained by the fact that in the crystal structure, DNA is only in direct contact with loop 1 residues of one monomer. (C) Regions with decreased root mean square fluctuations (RMSF) color coded on the MetJ-dna structure. Colors range from blue for no change to red for strongly decreased fluctuations. Strong stiffening is observed for helix C (C′) and loop 1. Side-chain fluctuations of Glu39 and Arg42 are quenched due to direct interaction with SAM (zoom), whereas the stiffening of Arg40 is an indirect effect, compare to Fig. 3D. Stiffening spreads to large parts of helix A. (D) The most dominant mode of fluctuation derived from MD simulations of MetJ-dna mapped on a cartoon representation. The first three eigenvectors were used to generate the trajectory. The two overlaid structures show the extreme positions when projecting along these eigenvectors. Amplitudes of fluctuations were exaggerated for better visibility. Loop 1 and helix C are marked red. (E) The network propagating fluctuations between helix C and loop 1. PCA on residue averaged pair-wise forces 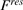 for apo MetJ-dna reveals a network of coupled interactions (see Methods). Edges represent residue pairs for which the first eigenvector is 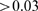. Edges within the first monomer are colored blue, those within the second red.

The loops formed by residues 12–20 (referred to as *loop 1*) suggest themselves to be involved in the allosteric mechanism, as they strongly differ in conformation between the 1CMC (MetJ) and 1CMA (MetJ-dna) crystal structures and are in direct contact with the DNA, Fig. 1C. NMR data for these loops shows a strong quenching of ns time-scale fluctuations upon co-repressor binding (Steve Homans, Leeds University, 2009, personal communication). In good agreement with these experimental findings our simulations of the MetJ-dna system show a strong decrease in backbone RMSF for loop 1 residues upon SAM binding, as well as stiffening of helix C, Fig. 5A–C. Quenching is observed for both the MetJ and MetJ-dna system, though less pronounced for the former (see below). Remarkably, principal component analysis (PCA) on the trajectory data reveals the dynamics of the distal loop 1 and helix C regions to be highly coupled, Fig. 5D, and the dynamics of both MetJ monomers to be highly cooperative. The lowest frequency mode (Eigenvectors 1–3) for apo and holo structures of MetJ-dna describe highly similar fluctuations, yet at very different amplitudes. Strong quenching of fluctuations is reflected by a decrease of the highest Eigenvalue from 120 (apo) to 28 (holo), Fig. S1. These observations are supported by entropy calculations based on Schlitter's formula [32]. Upon SAM binding, we find a decrease in entropy of 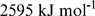 for MetJ-dna and 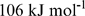 for MetJ, see also Table 1. The quantitatively different, yet qualitatively equivalent, results might be caused by the different crystal structures used, i.e the differences for loop 1 and adjacent residues. Overall, we find the stiffening effect of SAM to be independent from the presence of DNA.

**Table 1:** Changes in entropy upon co-repressor binding.

	Protein	Protein+DNA	DNA
MetJ-APO	54256	—	—
MetJ-SAM	54150	—	—
MetJ-dna-APO	57060	66149	9319
MetJ-dna-SAH	55508	64427	9083
MetJ-dna-SAM	54465	63341	9023

All values were calculated using Gromacs-4.0.5, units are in 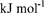.

The question arises how the distal helix C and loop 1 regions are dynamically linked through a largely rigid core of the MetJ-DNA system. To elucidate the communication pathway, we performed PCA on residue averaged pair-wise forces, 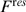, here termed *force-PCA*. Again, observing forces directly has the unique advantage to allow for following the complete propagation pathway, including parts showing only subtle coordinate changes. Force-PCA on MetJ-dna revealed a network of correlated changes in pair-wise forces, Fig. 5E. The network spans through the protein core, linking helix C and loop 1, the latter of which is connected to the rest of the network via residues Tyr11, Ile28, Lys31 and Glu55. Synchronization of the fluctuations between both monomers is achieved by force propagation along helix A and the 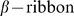. We found the allosteric signal caused by SAM binding to target large parts of helix A, in particular Glu39, Arg40 and Arg42, resulting in wide-spread stiffening, Fig 4C. Helix A accounts for a large part of the network propagating fluctuations, moreover it directly is part of the link between helix C and loop 1, Fig. 5E. In summary, SAM binding alters correlated forces linking loop 1 and helix C thus affecting the dynamics of these regions.

### Differential regulatory effect of SAM and SAH

The SAM analogue SAH has no regulatory function, i.e. no impact on the MetJ activity for binding to DNA, yet has the same binding mode and similar binding affinities as SAM (S. Philipps, Leeds University, 2009, personal communication). Based on this observation, an entirely electrostatic activation of MetJ by the positively charged SAM has been suggested [27]. We decided to elucidate differences between SAM and SAH binding, and to this end performed simulations of MetJ-dna in complex with SAH as co-repressor. We modeled the MetJ-SAH structure by removing the 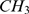 group from the sulfur atom of SAM in the 1CMA crystal structure used as template.

The overall conformation of MetJ-dna is not affected when replacing SAM by SAH, both structures have a backbone RMSD of only 0.42 Å. Also, the potential energy between protein and DNA 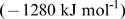 is quasi identical to the energy measured for MetJ-SAM and DNA 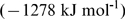. As for the co-repressor, our simulations show strong quenching of fluctuations upon SAH binding, yet quenching is less distinct. This is reflected by higher backbone-RMSF for MetJ-SAH throughout the protein, Fig, 5B, as well as a higher eigenvalue of 48 for the first eigenvector, what is significantly above 28, the value measured for SAM. Both eigenvectors describe a very similar mode of fluctuation, Fig. S1C. The flexibility of the bound ligand itself is increased as well. We measured an almost twofold increase in RMSF for SAH when compared to SAM (0.89 Å vs. 0.57 Å), apparently due to the loss of backbone interactions with SAM's positive charge.

**Figure 6:**
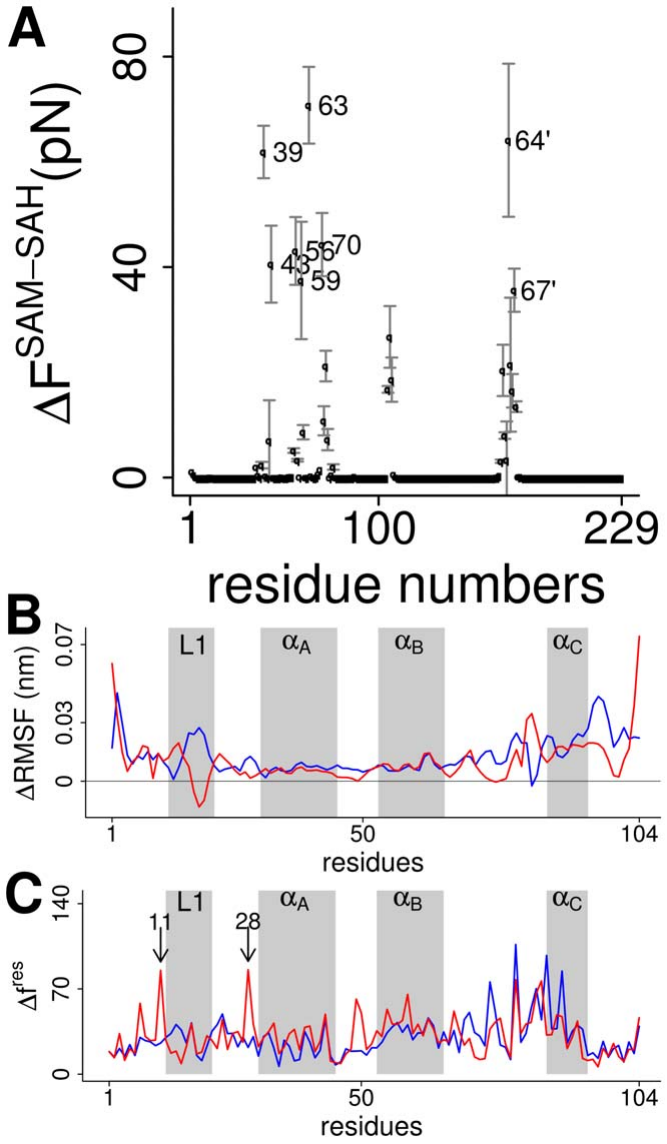
Differences between SAM and SAH. (A) Changes in residue-wise forces 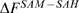 for MetJ-dna when replacing SAM by SAH. As expected, the strongest differences are observed for residues in close proximity to the charged sulfur atom. (B) Increased quenching of dynamics upon SAM binding. Plotted are differences in backbone RMSF for MetJ-dna in complex with SAM and SAH along the protein sequence for both monomers (red and blue). Positive values indicate increased stiffening for MetJ-SAM. The secondary structure is marked in gray. (C) Difference in residue wise forces 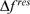 for MetJ-dna when substituting SAM by SAH for both monomers (red and blue). The secondary structure is marked in gray. Tyr11 and Ile28 (marked with arrows) show a high 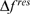 in the second monomer for which the DNA is fully resolved in the crystal structure.

Indeed, and unsurprisingly, the changes in direct interactions between the co-repressor and individual residues are significant, Fig. 6A. Removing the positive charge alters the charge distribution of SAM's whole methionine group, and we see changes in interaction even for residues as far as in helix A (residues 39 to 43), though most of the observed changes affect residues in direct proximity to the sulfur atom (residues 59 to 67). These changes lead to wide-spread alterations in the overall force propagation pattern, which are most pronounced in helix C and the proceeding loop, Fig. 6C. Interestingly, we find high changes in forces for Tyr11 and Ile28, both of which were found to link fluctuations of loop 1 with helix C by force-PCA. However, this effect is only present in the domain with the full DNA fragment resolved (residues 106–209 in the 1CMA structure), and thus further validation is necessary.

As the differences in binding affinity between SAM and SAH are of primarily entropic nature, we performed entropy calculations on MetJ-dna based on Schlitter's formula [32]. Vibrational entropies were calculated on the whole trajectory data totaling 300 ns per system and are sufficiently converged to allow semi-quantitative comparisons between SAM and SAH, Fig. S3. We found an entropy difference of 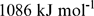 between SAM and SAH as co-repressor, of which the protein dynamics with 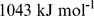 accounts for the major contribution. All values are given in Table 1. The absolute conformational entropies of 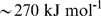 (apo) and 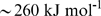 (holo) per residue are in agreement with previous estimates for other proteins [33],[34]. The values clearly show that there is a significant increase in entropy when substituting SAM by SAH, consistent with the observed difference in regulatory function. Both, the overall RMSF and the entropies suggest SAM to reduce MetJ flexibility more efficiently than SAH.

## Discussion

We have analyzed force distribution and dynamics in MetJ, a stiff allosteric protein regulated by SAM, its co-repressor. FDA allowed us to identify the network of interactions guiding force modulation within MetJ by cofactor binding. Experimental data, among others the inactivity of SAH as a co-repressor, suggest that a long range electrostatic interaction between DNA and the positive charge on SAM may exclusively explain MetJ activation [35]. Notwithstanding, there is evidence from mutagenesis experiments that charge alone cannot explain MetJ activation [28]. We here suggest strain propagation by subtle alterations of the MetJ structure as an important mode of allosteric signal propagation. The highly anisotropic distribution of internal strain leads to conformational re-adjustments at the interaction interface, mainly of Glu39, Arg40, Arg42, Arg43 and residues 50–53. Our simulations thus predict adjustments of these specific protein-DNA interactions to be an important factor for efficient DNA binding. Such a mechanism would allow MetJ to easily move along or between DNA strands until the target side is found, thereby speeding up target site location as recently proposed [36].

While the importance of this communication pathway has been experimentally probed by the loss of allosteric function upon mutation of residues identified as key residues by FDA, it is independent of the positive charge on SAM, as we find it similarly for SAH. This pathway therefore apparently causes or is complemented by an additional allosteric mechanism unique to SAM. We find the major SAM-dependent allosteric function of MetJ to come from an entropic contribution due to quenching of slow backbone and fast side chain dynamics. Only for SAM, the force network communicating the allosteric signal between loop 1 and helix C can substantially reduce correlated fluctuations. This is supported by theoretical models [37] as well as NMR data that suggest dynamics to play an important role (Steve Homans, Leeds University, 2009, personal communication). The major correlated motion that is quenched involves parts distant to each other as well as to the co-repressor binding site. Again, measuring correlated forces instead of coordinates revealed the role of the protein core in this long-range communication and allosteric regulation. We find a strong increase in entropy when substituting SAM by SAH, suggesting that the regulatory difference between SAM and SAH is of entirely entropic nature. It is the differential effect of SAM and SAH on the correlated forces involved in this motion that is likely to be responsible for the observed difference in allostery.

Dynamics are increasingly revealed as a regulatory driving force [38]–[40] and have recently been found for another transcription factor, the CAP protein [9]. We here find a similar mechanism for MetJ, suggesting that changes in dynamics upon cofactor binding may be a commonly used regulation pattern. Long-range allostery in the absence of any noticeable conformational change as featured by MetJ has remained a challenge for structure-based experimental and theoretical approaches. In combination with conventional analysis of the MetJ dynamics, we find FDA an optimal tool to track an allosteric pathway in MetJ. Signal propagation was found to be largely hidden in unremarkable shifts in atomic coordinates. Yet, these mere conformational shifts, as revealed by FDA, can involve large changes in forces for strongly interacting atom pairs, resembling “stiff springs” in the protein interaction network. Monitoring forces instead of coordinates therefore renders FDA highly sensitive. Pure conformational analysis would simply overlook rearrangements of the magnitude reported here, especially as properties such as root-mean square deviations or fluctuations are easily dominated by slow sub-domain movements, as it is the case for MetJ, Fig. 5C. By considering pair-wise forces which are, by definition, dominated by strong and relatively short-ranged interactions, such large fluctuations have only minor influence. Pair-wise interactions have the additional advantage of being independent from any fitting scheme, as conventionally used for RMSD or RMSF calculations, thereby not introducing any bias by the arbitrary choice of a reference structure. The same multivariate statistical methods, such as PCA, that are used for the analysis of coordinate based trajectory data can be applied to pair-wise forces. Again, one has the advantage of being able to observe relations that would otherwise be below the sensitivity of the method.

We recently determined the force bearing scaffold in a titin immunoglobulin domain, a protein mainly designed to withstand mechanical load by means of FDA [16]. Here, we present the first successful application to a stiff allosteric protein, opening the road to better understand the function of a whole class of proteins, including enzymes, by examining their internal force network. We note that FDA does not require extensive sampling of an allosteric conformational transition, which at current simulation time-scales is out of reach for most proteins. This is an unique advantage over other MD based simulation techniques used for studying protein allostery. FDA is content with monitoring the development of internal strain prior to the eventual shift in the protein conformational ensemble. We predict forces averaged over a total simulation time in the sub-microsecond range to suffice for the analysis of much slower allosteric signaling pathways. Importantly, while we here modified the Gromacs simulation suite to add FDA functionality, virtually any MD simulation package can be easily modified to include FDA at practically no additional computational expense, as pair-wise forces are anyways calculated at each time step.

Our results highlight the strength of FDA as a tool supporting experimental design, as it can straightforwardly be verified by experimental studies. In particular, our results suggest Arg40, Thr37 and Asn53 at the MetJ-DNA interaction interface to be important for allosteric function. Mutations of Arg40 and Thr37 have indeed been previously shown to abolish SAM-dependent allosteric regulation of MetJ [31]. In addition, we predict mutation of Glu59 and Arg43, forming the salt bridge between helix A and B, and the crucial SAM interaction partners Glu39 and Arg42 to lower the co-repressor activity of SAM.

## Methods

### 

#### Molecular dynamics simulations

All simulations were carried out using Gromacs 4.0.4 [41]. The Amber03 all atom force field [30] for the protein and the TIP3P [42] water model were employed. Crystal structures of the MetJ holo form (PDB-entry 1CMC) and MetJ in complex with DNA (PDB-entry 1CMA) were used as starting structures for all simulations. Protonation states of histidines were determined by optimizing the hydrogen bond network using Whatif [43]. MetJ apo forms, with and without DNA, respectively, were created by deleting SAM from the crystal structures. The structure containing SAH as co-repressor was derived from the 1CMA structure by removing the 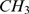 group from the sulfur atom of SAM. Structures including crystal waters were solvated in a cubic box of size 93 Å containing 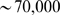 atoms. Sodium and chloride ions corresponding to a physiological ion strength of 100 mM were added. Negative charges on the DNA were compensated by adding additional sodium ions, which we found to preferentially locate around the DNA, as expected, Fig. S4. An energy minimization of 1000 steps using the steepest descent algorithm was followed by a 500 ps MD simulation with harmonic restraints on the protein heavy atoms with a force constant of 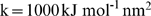 to equilibrate water and ions. A subsequent free MD simulation of 6 ns length was performed to equilibrate the whole system, during which the protein backbone root mean-square deviation (RMSD) was monitored. Both structures remained highly stable, with a backbone RMSD to the starting structure 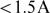.

Forces and average coordinates were then obtained from 10 independent 30 ns equilibrium simulations for each configuration. For each simulation new random velocities were generated and a new starting frame from the last 3 ns of the equilibration run was chosen to ensure optimal conformational sampling. Simulations were run in the NpT ensemble. Temperature was kept constant at 300 K by coupling to the Nose-Hoover thermostat [44]. The pressure was kept constant at 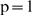 bar using anisotropic coupling to a Parrinello-Rahman barostat [45] with 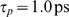 and a compressibility of 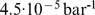 in the x, y, and z directions. All bonds were constrained using the LINCS [46] algorithm; an integration timestep of 2 fs was used. Lennard-Jones interactions were calculated using a cutoff of 10 Å. At a distance smaller than 10 Å, electrostatic interactions were calculated explicitly, whereas long-range electrostatic interactions were calculated by Particle-Mesh Ewald summation [47]. System coordinates were saved every 2 ps.

Potential energies were calculated using Gromacs. Energies are averages over the whole simulation time and only included non-bonded interactions below the cutoff distance. SAM and SAH were excluded from the energy calculations.

#### Force distribution analysis

We used the FDA code [16] for Gromacs-4.0.4 to write out forces 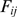 between each atom pair 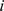 and 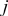 as calculated during our MD simulations. During each step of an MD simulation forces between all atom pairs within a cutoff range are calculated. These vectorial forces are then summed up in order to calculate the acceleration on each atom, and average to zero over time. The FDA code instead writes these pair-wise forces out prior to summation. Since pair-wise force vectors are subject to change upon rotation and translation of the system, we use the norm of the force acting between each atom pair, with opposite signs assigned to attractive and repulsive forces. This allows to calculate time-averaged forces, which measure how much strain the interaction is carrying.

Forces were averaged over accumulated simulation times of at least 300 ns per system in order to arrive at converged averages. Changes in forces, 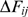, were then obtained as the difference in pair wise forces between the MetJ in holo and apo form. To remove outliers, i.e. some large solvent exposed side chains showing a high 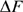 due to insufficient conformational sampling, we normalize forces with the standard error between individual trajectories as described before [16]. Changes in normalized force are denoted 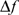. Residue wise forces 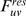 were obtained by summing up forces 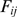 for all pairs of residues 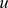 and 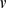, where atom 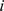 and atom 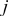 must not be part of the same residue; normalization was done as for inter atomic forces. The absolute sum 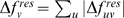 reflecting the change seen by a single residue, was used to calculate correlations between residue wise forces in MetJ and MetJ-dna and to map changes in 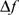 onto the protein structures.

Forces include contributions of individual bonded (angle, dihedral) and non-bonded (electrostatic and van der Waals) terms below the cutoff distance, which are stored separately to allow independent analysis. Due to the use of LINCS no forces for bonds could be calculated. The force between each atom pair is represented as the norm of the force vector and thus is a scalar. Attractive and repulsive forces are assigned opposite signs. As we consider the direct force between each atom pair, the equilibrium force can be different from zero, even for the theoretical case of a system without any motion and in the absence of an external perturbation. Monitoring changes in pairwise forces instead of atomic displacements has the advantage of observing signal propagation even through stiff materials [17], where forces propagate without causing major atomic displacement.

#### Principal component analysis and entropy calculations

PCA on the trajectory data was carried out based on the mass-weighted covariance matrix of atomic coordinates, as calculated by Gromacs. Eigenvalues and eigenvectors were calculated by diagonalizing the covariance matrix, and eigenvectors were sorted in descending order of their eigenvalue. Modes of fluctuation were visualized by calculating a trajectory along the first three eigenvectors. In all systems, most of the observed covariance is already captured within the first three eigenvectors, as reflected by the eigenvalue structure, Fig. S1. Entropy calculations were performed based on the covariance matrix using the quasi-harmonic approximation (Schlitter formula [32]) as implemented in Gromacs.

FPCA was done on a trajectory of of residue averaged pair-wise forces, 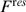. The trajectory contained forces between all residue pairs during a 30 ns simulation. An output frequency of 20 ps was used, totaling 1500 data points per residue pair. Including all possible residue pairs into the calculation of the covariance matrix would lead to a matrix of size 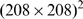, what is computationally not feasible. We thus only included columns with a mean force 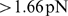, what lead to a covariance matrix of dimension 3422. PCA was done individually for the x, y and z components of the residue wise forces, again most of the observed covariance could already be captured within the first eigenvector. A cumulative eigenvector consisting of the first eigenvectors of the x, y, z components was created by calculating the norm of all (x, y, z) triplets, this eigenvector was used to draw edges in Fig. 5. All FPCA calculations were done in R [48].

#### Parameterization of SAM and SAH

SAM consists of an adenosyl group, and a methionine linked together, Fig. 1B. Bonded parameters for the adenosyl group, methionine and the linkages between them are available in the Amber03 [30] force field; parameters for SAM's charged sulfur atom were adopted from the generalized amber force field [49]. We used quantum chemical (QM) calculations to calculate ESP charges on the SAM/SAH atoms and all QM calculations were carried out using Gaussian03 [50]. In the uncharged SAH molecule the sulfur atom forms two covalent bonds with the adjacent carbons, whereas SAM forms a third covalent bond with an additional methyl group. Thus the overall charges in the QM calculations are zero for SAH and one for SAM. The B3LYP method [51],[52] in density functional theory combined with the correlation consistent basis set cc-pvtz (B3LYP/cc-pvtz) [53] as implemented in Gaussian was used to perform single energy calculations. We chose this method as it was used during development of the Amber force field and thus ensures maximal compatibility. ESP charges were calculated by fitting to the electrostatic potential at selected points according to the Merz-Singh-Kollman scheme [54],[55]. The solvation effect is considered implicitly by use of the polarizable continuum mode (PCM [56],[57]) at a temperature of 298 K. The value of the dielectric constant of the PCM model is set to 4 to mimic an environment inside a protein. Charges derived from our calculations are in good agreement with the charges used in the Amber force field. The full parameter set is available as Dataset S1.

## Supporting Information

Figure S1 Stiffening and modes of fluctuation in MetJ. (A) The sum of the three eigenvectors with largest eigenvalue for apo and holo MetJ-dna plotted against each other; the correlation coefficient is R = 0.71. The line shows the fit of the data to a linear model. The corresponding eigenvalues are plotted below, with blue for apo and red for holo MetJ-dna. The high similarity between the eigenvectors indicates that the principal mode of fluctuation is only slightly affected by SAM binding. However, the amplitude of the fluctuation, given by the eigenvalues, is decreased almost 5 fold. This indicates strong quenching of fluctuations, as was also measured in terms of decreased RMSF. (B) The sum of the three eigenvectors with largest eigenvalue for apo and holo MetJ plotted against each other; the correlation coefficient is R = 0.78. The line shows the fit of the data to a linear model. The corresponding eigenvalues are plotted below, with blue for apo and red for holo MetJ. (C) The first three eigenvectors for MetJ-dna bound to SAM and SAH are highly similar. Plotted are the sums of the three eigenvectors with largest eigenvalue for MetJ-dna in complex with SAM and SAH against each other. The line shows the fit of the data to a linear model. (D) The modes of fluctuation are highly similar for MetJ and MetJ-dna. Plotted are the sums of the three eigenvectors with largest eigenvalue for MetJ and MetJ-dna against each other (apo and holo configuration). The line shows the fit of the data to a linear model.
(0.38 MB TIF)

Figure S2 Similarity of the force distribution pattern of the individual dimers. (A) Plotted are residue wise forces Δf^res^ of one MetJ homodimer against the other. MetJ shows a highly symmetric force distribution pattern, with correlation coefficient R = 0.83. In all plots the line shows a fit of the data to a linear model. (B) The force distribution pattern in MetJ-dna is less symmetric (R = 0.66), what might be due to the lower resolution of the crystal structure or the only partially resolved DNA. (C) Exchanging SAM by SAH has only minor effects on force distribution. Residue wise forces for MetJ-dna in complex with SAM and SAH correlate with R = 0.83.
(0.16 MB TIF)

Figure S3 Convergence of entropies calculated using Schlitter's formula. Plots show the convergence of the entropy with increasing simulation time. Shown are entropies calculated for apo and holo forms for (A) MetJ-dna, only the protein contribution (B) MetJ-dna, only DNA contribution (C) MetJ-dna, the Protein-DNA complex and (D) MetJ.
(0.38 MB TIF)

Figure S4 Ion distribution, measured in terms of the radial distribution function (RDF), around the DNA. The plot shows the average distribution of Na+ and Cl- ions around the DNA during 300ns. As expected, we found positively charged ions to accumulate around the DNA.
(0.09 MB TIF)

Dataset S1 Amber parameters for SAM and SAH. The zip archive contains the additional parameters added for sam, together with amber .prep and .pdb files of SAM and SAH.
(0.01 MB ZIP)
