# Supplementary figures and images for: Dynamic Allostery in the Methionine Repressor Revealed by Force Distribution Analysis
Source: PLoS Comput Biol. 2009 Nov 20;5(11):e1000574. doi: 10.1371/journal.pcbi.1000574 (PMC2775130; doi:10.1371/journal.pcbi.1000574)

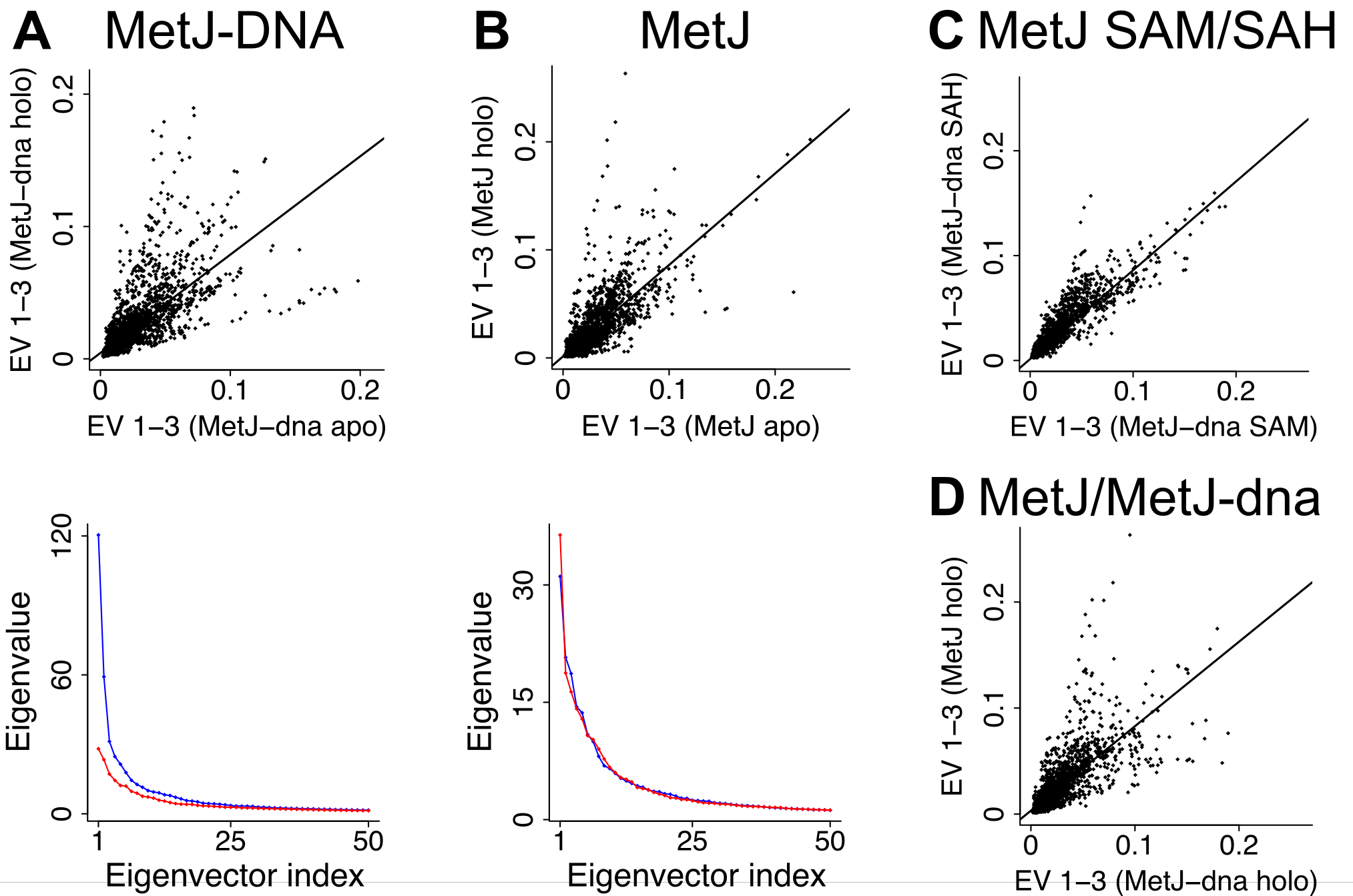

Supplement: Figure S1 — Stiffening and modes of fluctuation in MetJ. (A) The sum of the three eigenvectors with largest eigenvalue for apo and holo MetJ-dna plotted against each other; the correlation coefficient is R = 0.71. The line shows the fit of the data to a linear model. The corresponding eigenvalues are plotted below, with blue for apo and red for holo MetJ-dna. The high similarity between the eigenvectors indicates that the principal mode of fluctuation is only slightly affected by SAM binding. However, the amplitude of the fluctuation, given by the eigenvalues, is decreased almost 5 fold. This indicates strong quenching of fluctuations, as was also measured in terms of decreased RMSF. (B) The sum of the three eigenvectors with largest eigenvalue for apo and holo MetJ plotted against each other; the correlation coefficient is R = 0.78. The line shows the fit of the data to a linear model. The corresponding eigenvalues are plotted below, with blue for apo and red for holo MetJ. (C) The first three eigenvectors for MetJ-dna bound to SAM and SAH are highly similar. Plotted are the sums of the three eigenvectors with largest eigenvalue for MetJ-dna in complex with SAM and SAH against each other. The line shows the fit of the data to a linear model. (D) The modes of fluctuation are highly similar for MetJ and MetJ-dna. Plotted are the sums of the three eigenvectors with largest eigenvalue for MetJ and MetJ-dna against each other (apo and holo configuration). The line shows the fit of the data to a linear model. (0.38 MB TIF) [file pcbi.1000574.s001.tif]

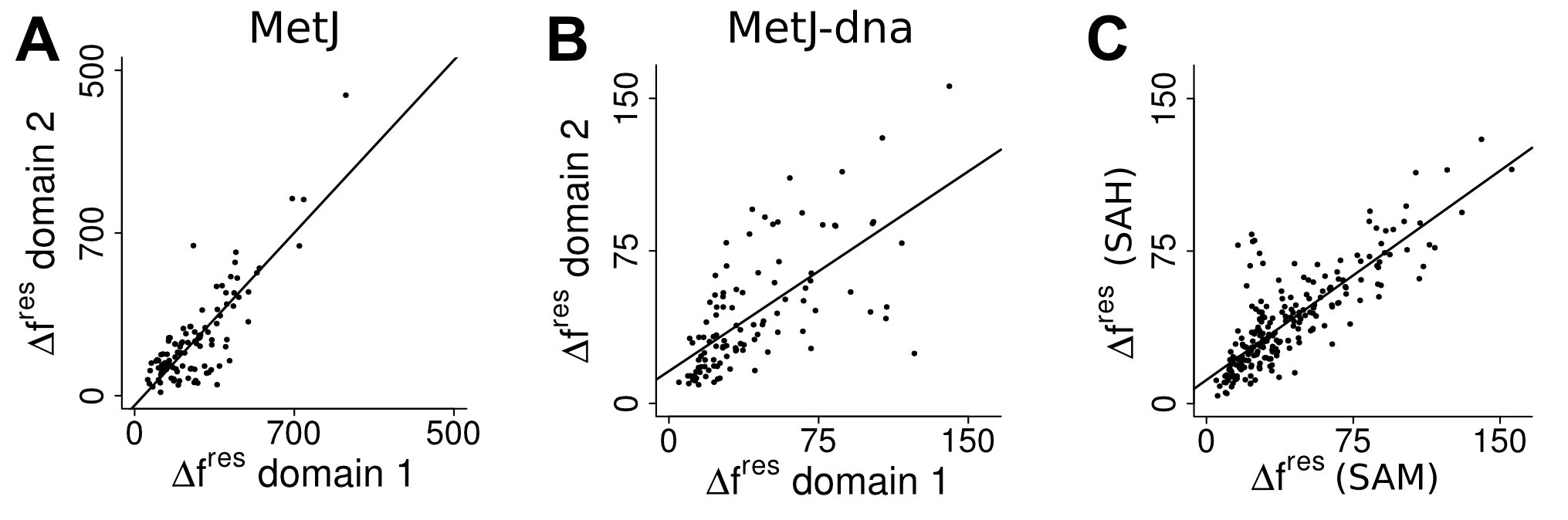

Supplement: Figure S2 — Similarity of the force distribution pattern of the individual dimers. (A) Plotted are residue wise forces Δfres of one MetJ homodimer against the other. MetJ shows a highly symmetric force distribution pattern, with correlation coefficient R = 0.83. In all plots the line shows a fit of the data to a linear model. (B) The force distribution pattern in MetJ-dna is less symmetric (R = 0.66), what might be due to the lower resolution of the crystal structure or the only partially resolved DNA. (C) Exchanging SAM by SAH has only minor effects on force distribution. Residue wise forces for MetJ-dna in complex with SAM and SAH correlate with R = 0.83. (0.16 MB TIF) [file pcbi.1000574.s002.tif]

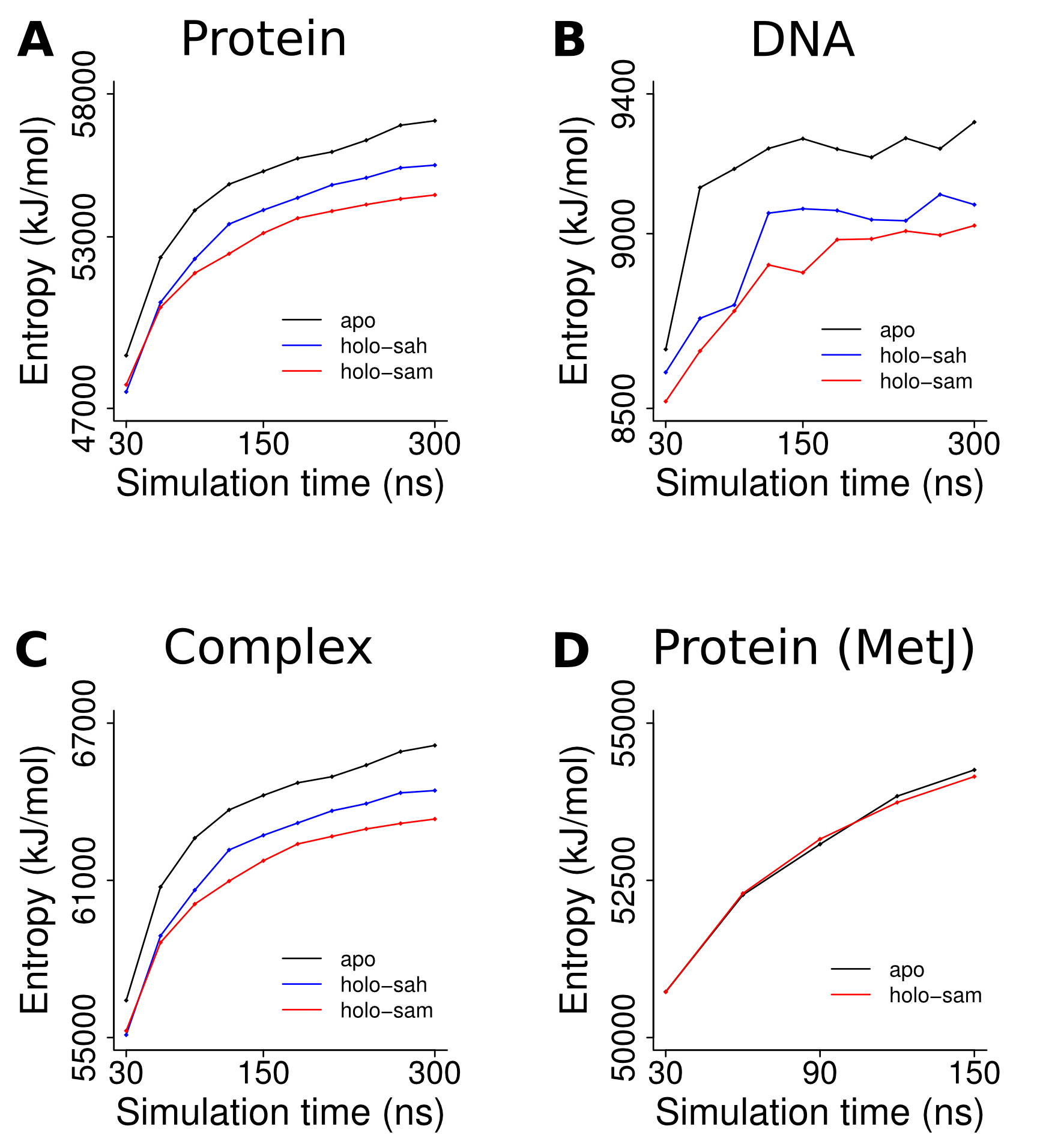

Supplement: Figure S3 — Convergence of entropies calculated using Schlitter's formula. Plots show the convergence of the entropy with increasing simulation time. Shown are entropies calculated for apo and holo forms for (A) MetJ-dna, only the protein contribution (B) MetJ-dna, only DNA contribution (C) MetJ-dna, the Protein-DNA complex and (D) MetJ. (0.38 MB TIF) [file pcbi.1000574.s003.tif]

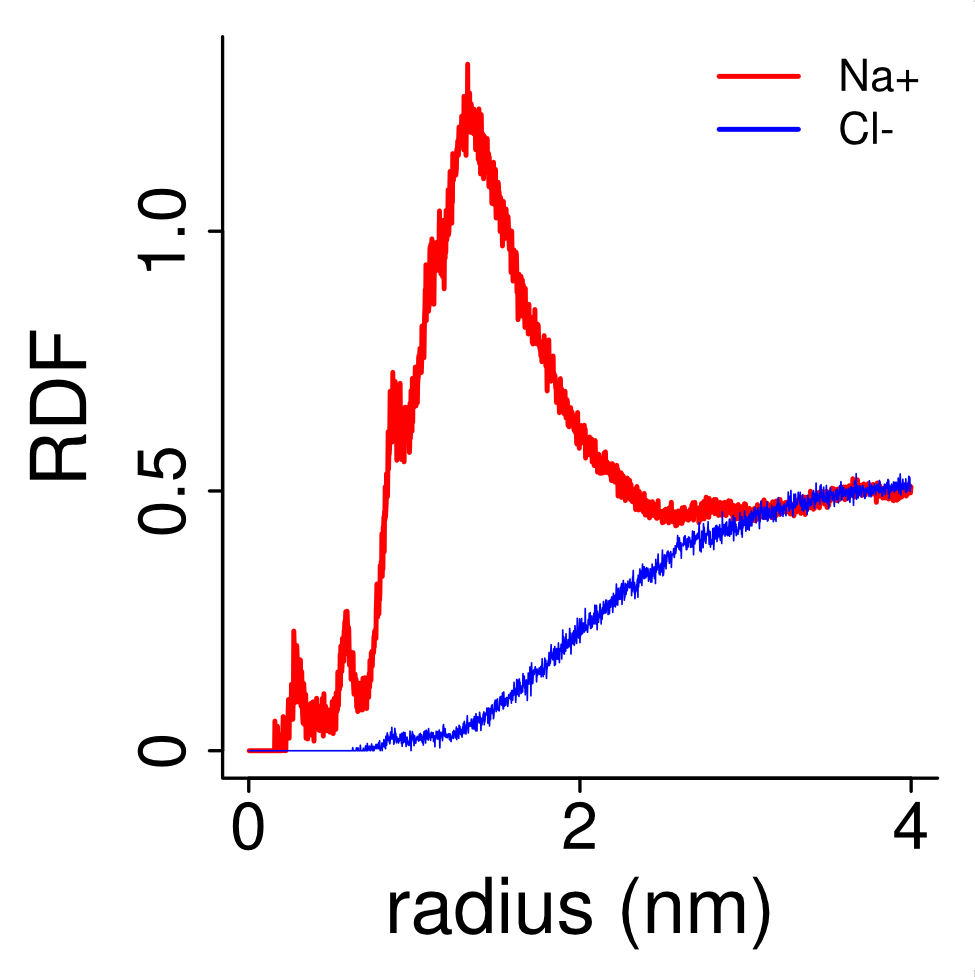

Supplement: Figure S4 — Ion distribution, measured in terms of the radial distribution function (RDF), around the DNA. The plot shows the average distribution of Na+ and Cl- ions around the DNA during 300ns. As expected, we found positively charged ions to accumulate around the DNA. (0.09 MB TIF) [file pcbi.1000574.s004.tif]
